# Taking the Impression

**Published:** 1906-05

**Authors:** Stewart J. Spence

**Affiliations:** Chattanooga, Tenn.


### TAKING THE IMPRESSION.

Stewart J. Spence, D. D. S., Chattanooga, Tenn.

*Preparation of mouth.*—Determine (1) the condition of the mucous membrane, and (2) of the remaining teeth, if any. If only a few teeth remain in the upper jaw, it is better to remove them, especially if loose and straggling, because they are not needed for clasping since sufficient adhesion can almost always be obtained for upper plates, and they may prevent the correct adjustment of the porcelain teeth. Their tendency also to become sensitive if the cementum is exposed, and to decay at points in proximity with the plate, and the tendency to gingivitis, cautions extraction. On the contrary, if lower teeth remain they should be preserved, if possible, because of their great usefulness as supports to the plate. This applies only to the eight lower anterior teeth, and especially to the first bicuspids. If second bicuspids or molars remain in a badly decayed or loose condition, they should be extracted. A straggling molar or two, if left, necessitate so small a saddle surface of plate, and also interfere so seriously with correct articulation, as to be better out than in; and loose bicuspids are of little service for clasping.

Where full upper plates have been worn in connection with only the lower anterior teeth, the membrane under the plate in the upper incisor region will often be found flabby. As this condition will prevent stability of the new plate in biting, said membrane should be cocainized and freely removed by scissors.

Where tartar is found, remove it thoroughly. Grind down sharp edges of enamel and teeth that are larger than their companions. Beware of excessive grinding, for obstinate odontalgia may result. Touch nitrate of silver to the freshly ground parts.

*Taking the impression.*—Where a full upper impression can be correctly taken in modeling compound this material is best, because it has a slight contraction, which tends to throw bearing of plate on ridge, not on roof. But if, in taking it, pressure is made on first one side of tray then the other, this material will be compressed a little on each side by each such pressure, and as it will not, as will plaster, flow back again there will result a space between compound and membrane along ridge. To avoid this, hold firmly and steadily with both hands, but as near to the centre of tray as possible. The nearer the centre the less the leverage.

For partial lower impressions use plaster, because of the dragging of modeling compound, which is liable to affect more than the part immediately around teeth. If, however, the compound be used after loosening and partly removing it, press it back for a minute. When plaster is used for impression, mix with it about one-sixth marble dust, or pumice, to cause soft-setting and so allow easy breakage and removal from around teeth, both in removing impression from mouth and model from impression.

To avoid the expansion of plaster of Paris, use the finest grade and stir as little as possible; then pour model into impression as soon as possible; for most Paris plasters do not get their full expansion when short stirred, in less than thirty minutes. Better use Spence's non-expanding plaster.

Try tray in mouth, and if necessary bend or cut it to shape, or build it up with baseplate wax. Most lower trays need to be cut away at their posterior buccal corners.

For upper impression, stand behind patient, hold handle of tray in line with nose, and press posterior part of tray somewhat in advance of the anterior, in order that excess, if any, may be pushed forward. If high palate, first fill same with a little plaster, thus avoiding air spaces.

Paint soft palate with solution of cocaine before taking impression.

Wipe the mouth clean of mucus with soft cloth immediately before taking impression. If impression comes out covered with mucus, better retake it.

For lower impression, stand before patient, with chair elevated. On inserting, push back with fingers the cheeks, folds of which are apt to get under the impression. Then ask patient to raise tongue, and then press down lightly. If lower incisors remain, insert finger and press plaster well against them and subjacent surface. Then press down firmly and hold steadily till hard.

Lower impressions should be filled with plaster in lingual space, so that the model will be strengthened by being bridged across said space, not horse-shoe shaped.

